# Interleukin-10 Deficiency Impacts on TNF-Induced NFκB Regulated Responses In Vivo

**DOI:** 10.3390/biology11101377

**Published:** 2022-09-20

**Authors:** Stamatia Papoutsopoulou, Liam Pollock, Jonathan M. Williams, Maya M. L. F. Abdul-Mahdi, Reyhaneh Dobbash, Carrie A. Duckworth, Barry J. Campbell

**Affiliations:** 1 The Henry Wellcome Laboratories of Molecular & Cellular Gastroenterology, University of Liverpool, Liverpool L69 3GE, UK; 2 Department of Biochemistry and Biotechnology, School of Health Sciences, University of Thessaly, 41500 Larissa, Greece; 3 Department of Molecular Physiology & Cell Signalling, Institute of Systems, Molecular and Integrative Biology, University of Liverpool, Liverpool L69 3GE, UK; 4 Pathobiology and Population Sciences, Royal Veterinary College, Hatfield AL9 7TA, UK; 5 School of Life Sciences, University of Liverpool, Liverpool L69 3GE, UK; 6 Department of Infection Biology & Microbiomes, Institute of Infection, Veterinary and Ecological Sciences, University of Liverpool, Liverpool L69 3GE, UK

**Keywords:** interleukin-10, intestine, mucosa, NFκB, RelA, tumour necrosis factor

## Abstract

**Simple Summary:**

Chronic inflammation of the gut is a multifactorial, incurable condition that involves interactions between our immune cells and the cells that line the surface layer of gut, known as the epithelium. Interleukin-10, a protein well known to be released by our immune cells, has a protective role in countering inflammation, partly due to the fact that it can block the NFκB pathway, a signalling pathway within cells that promotes genes involved in inflammatory responses. However, we recently showed that laboratory cultures of gut epithelial cells can also synthesise interleukin-10, which acts as a positive regulator of the NFκB pathway to support gut health. In this study here, we investigated further the impact of the role of interleukin-10 on the NFκB pathway and its targets within the gut using a whole animal approach, and confirmed that NFκB activation is indeed positively regulated by interleukin-10, affecting the expression of downstream target genes and their encoded proteins. This strengthens the importance of the interleukin-10/NFκB signalling pathway axis in maintenance of gut health and response to damage, inflammation, and infection. Understanding cell-specific biological roles of interleukin-10 and its interactions with NFκB could prove useful for future therapeutic intervention for interleukin-10 regulated inflammatory conditions.

**Abstract:**

Interleukin-10 (IL-10) is an anti-inflammatory cytokine that has a major protective role against intestinal inflammation. We recently revealed that intestinal epithelial cells in vitro regulate NFκB-driven transcriptional responses to TNF via an autocrine mechanism dependent on IL-10 secretion. Here in this study, we investigated the impact of IL-10 deficiency on the NFκB pathway and its downstream targets in the small intestinal mucosa in vivo. We observed dysregulation of TNF, IκBα, and A20 gene and protein expression in the small intestine of steady-state or TNF-injected *Il10^−/−^* mice, compared to wild-type C57BL6/J counterparts. Upon TNF injection, tissue from the small intestine showed upregulation of NFκB p65[RelA] activity, which was totally diminished in *Il10^−/−^* mice and correlated with reduced levels of TNF, IκBα, and A20 expression. In serum, whilst IgA levels were noted to be markedly downregulated in IL-10-deficient*^-^* mice, normal levels of mucosal IgA were seen in intestine mucosa. Importantly, dysregulated cytokine/chemokine levels were observed in both serum and intestinal tissue lysates from naïve, as well as TNF-injected *Il10^−/−^* mice. These data further support the importance of the IL-10-canonical NFκB signaling pathway axis in regulating intestinal mucosa homeostasis and response to inflammatory triggers in vivo.

## 1. Introduction

Interleukin-10 (IL-10) is an anti-inflammatory cytokine that plays an important role in mucosal homeostasis [1]. In the healthy intestinal mucosa, IL-10 is produced by a wide variety of cell types, including intraepithelial lymphocytes (IEL) [2], a subdivision of type 2 innate lymphoid cells found within the lamina propria [3], dendritic cells, macrophages [4,5], intestinal fibroblasts, and intestinal epithelial cells [6,7]. IL-10, amongst other cytokines, promotes the differentiation of immunoglobulin A (IgA) producing plasma cells within the gut [8]. These IgA-producing plasma cells also play a key role in homeostasis and barrier protection of the gastrointestinal mucosa [9,10]. In the absence of IL-10, mice have been shown to develop age-related enterocolitis, which is associated with the dysregulation of lymphocyte populations, including an increased frequency of B1 cells, decreased numbers of Tregs, and an increased number of activated T cells in the intestine. [11]. Most IL-10-producing cells also express an IL-10 receptor (IL-10R), and the importance of this receptor in the maintenance of intestinal homeostasis and protection against development of intestinal inflammation, is now well established by several independent studies in macrophages [12,13] and T cells [14]. Transgenic mouse strains deficient in IL-10 or IL-10R are susceptible to develop spontaneous colitis early in life, which can be reversed by treatment with recombinant IL-10 [15,16]. Similarly, in humans, defective IL-10/IL-10R signaling has been shown to correlate with inflammatory bowel disease (IBD) [17,18]. Genome-wide association studies (GWAS) have also identified polymorphisms in *IL10* and *IL10R* genes, both associated with an early onset of IBD, a greater severity of disease, and a lack of response to conventional treatments, including anti-tumour necrosis factor (TNF) biologics therapy [19,20].

Downstream IL-10R signaling leads to the activation of the JAK/STAT (Janus kinases/signal transducer and activator of transcription proteins) pathway [21]. Importantly however, IL-10 also affects the nuclear factor kappa B (NFκB) transcription factor pathway, a key signaling pathway with an established role in the mediation of inflammation and a response to tissue damage and/or infection [22]. NFκB activation is also essential for maintenance of intestinal barrier integrity, through regulation of cellular proliferation, differentiation, and survival, and the mediation of signaling and interaction between the mucosal immune system and the resident gut microbiota [23]. Recent work from our own lab, utilising murine small intestine crypt stem-cell-derived 3D enteroid cultures, has demonstrated that NFκB activation is the predominant signal pathway response seen within the first hour following exposure to TNF [24]. We were also able to demonstrate that enteroids deficient in IL-10 show a defective activation of NFκB, with dysregulated gene expression of known downstream targets of NFκB, such as *Tnf*, and NFκB pathway inhibitor genes *Nfkbia* and *Tnfaip3*, encoding IκBα and A20 (tumor necrosis factor, alpha-induced protein 3, TNFAIP3), respectively [24]. Although our previous data clearly demonstrated that endogenous IL-10 may act as a positive regulator of the canonical NFκB pathway in vitro, we looked to provide further supporting evidence using a whole animal approach.

In this study, hence, we examined the effect of IL-10 deficiency on NFκB activation, and NFκB-dependent gene and protein expression within the intestinal epithelium, both in naïve mice and in animals following intraperitoneal injection with TNF. Our in vivo data have revealed that IL-10-deficient mice are characterized by systemic defects in NFκB-regulated serum cytokines and IgA, as well as defective NFκB p65[RelA] activity within the small intestine, which leads to a dysregulation of key NFκB target genes *Nfkbia* and *Tnfaip3* and expression of their encoded proteins.

## 2. Materials and Methods

### 2.1. Mice

Interleukin 10 knockout (*Il10^−/−^*) transgenic mice, developed by Kuhn and colleagues [15], established on C57BL/6J genetic background for several generations (B6.129P2-Il10^tm1Cgn^/J) by the Jackson Laboratory (Bar Harbor ME, USA; Stock No. 002251, www.jax.org/strain/002251; accessed on 12 August 2022), were purchased and imported via Charles River UK Ltd. (Margate, UK). Wild-type controls were age- and gender-matched C57BL/6J sub-strain mice, again provided by Charles River UK Ltd. Mice were bred and/or housed in a specific pathogen-free environment (Biomedical Services Unit; University of Liverpool, Liverpool, UK) and maintained on a regular diurnal lighting cycle (12:12 light:dark), with ad libitum access to irradiated CRM(P) pelleted chow (SDS Special Diet Services) and water. Studies involving animals were reviewed and approved by the Home Office of the United Kingdom under PPL number: PP5019347. At 10 weeks of age, the mice were treated with recombinant mouse TNF (Peprotech Ltd.; London, UK) diluted in sterile distilled water, administered via intra peritoneal (i.p.) injection at 0.33 mg/kg body weight. We have shown previously that TNF induces epithelial cell apoptosis and shedding 1.0 to 1.5 h post injection, as per [25]. We selected the 1.5 h time point to ensure induced responses within the intestinal mucosa, i.e., NFκB activation and induction of the NFκB inhibitors. Post injection of TNF (1.5 h), mice were euthanized by CO_2,_ as per Schedule 1 of the Animals (Scientific Procedures) Act 1986. Whole blood was collected by cardiac puncture from mice of both genotypes under resting and treatment conditions. Serum was isolated to measure levels of cytokines/chemokines and immunoglobulins by ELISA. Small intestinal tissue was dissected and snap frozen in liquid nitrogen to support qPCR, enzyme-linked immunosorbent assay (ELISA), and immunoblot studies. Additional tissue was fixed in formalin for immunohistochemistry (IHC) studies.

### 2.2. RNA Extraction and qPCR

Dissected tissues were disrupted in RLT buffer in a TissueLyser II (Qiagen; Manchester, UK) and total RNA was purified as per manufacturer instructions (RNeasy mini kit; Qiagen). Purified RNA was reverse transcribed using the High-Capacity RNA-to-cDNA Kit (Applied Biosystems; Paisley, UK), and cDNA was stored at −20 °C. qPCR reactions were performed in 96-well plates, with Taqman Fast advanced master mix (Applied Biosystems; Paisley, UK), Taqman Gene Expression Assay probes (Applied Biosystems), and 50 ng total cDNA as per manufacturer instructions. All qPCR reactions were carried out on a Roche LightCycler 480 (Roche; Basel, Switzerland), with conditions as follows: 1 cycle of: 120 s at 50 °C, 20 s at 95°C; 40 cycles of 3 s at 95 °C, 30 s at 60 °C, and 20 s at 60 °C; 1 cycle at 120 s at 72 °C and 30 s at 60 °C. Cp values were calculated from 2nd derivative analysis and relative quantification was calculated using 2-ΔΔCT method [26]. The gene expression assay probes used were *Tnf* (Mm00443258_m1), *Tnfaip3* (Mm00437121_m1), *Nfkbia* (Mm00477798_m1), *Il10* (Mm01288386_m1), *Il10ra* (Mm00434151_m1), *Tnip1* (Mm01288484_m1), and *Tnip2* (Mm00460482_m1). All results were normalized to the expression of the housekeeping gene *Gapdh* (Mm99999915_g1).

### 2.3. Immunohistochemistry

Formalin-fixed tissue was bundled using methods previously described [27]. Briefly, bundles were processed, embedded in paraffin wax in the transverse orientation, and 4 µm thick tissue sections were cut by microtomy and processed for immunohistochemistry (IHC). Tissue sections were de-paraffinised and rehydrated, then treated with 1% *v*/*v* hydrogen peroxide in methanol to block endogenous peroxidases, followed by heat-induced antigen retrieval in 0.01 M citrate acid buffer (pH 6.0). Sections were then blocked with 5% *v*/*v* serum supplied by respective ImmPRESS kits (Vector Laboratories, Heyford, UK), followed by primary antibodies against TNFα (rat monoclonal antibody MP6-XT22, Abcam; Cambridge, UK), TNFAIP3 (mouse monoclonal antibody 66695-1-Ig, Proteintech; Manchester, UK), and IκBα (rabbit polyclonal antibody 9242, Cell Signaling Technology; Leiden, The Netherlands). Secondary antibodies were from ImmPRESS polymer detector kits, and for TNFAIP3, the use of a mouse-on-mouse ImmPRESS kit. Slides were counterstained with haematoxylin (Vector Laboratories).

### 2.4. NF-κB p65[RelA] Activity ELISA

Small intestine tissue samples were homogenized in a 300 μL ice-cold radio-immunoprecipitation assay (RIPA) lysis buffer (Fisher Scientific; Loughborough, UK) for 20 min on ice followed by centrifugation at 10,000× *g* for 10 min at 4 °C, and supernatants were then stored at −80 °C. Lysates were assayed in duplicate, using a TransAm Flexi NF-κB ELISA kit for the activated form of p65[RelA] (Active Motif Europe; La-Hulpe, Belgium). Data were normalized against total cellular protein as measured in RIPA lysates by Pierce bicinchoninic acid (BCA) assay (ThermoFisher Scientific; Loughborough, UK).

### 2.5. ELISA for Serum and Tissue Cytokines, Chemokines and Immunoglobulins

Murine serum and RIPA-extracted small intestinal tissue lysates were analysed, as per manufacturer instructions, using the following ELISA kits: Cxcl1/KC (dy453-05; R&D systems; Abingdon, UK); Cxcl10/IP-10 (dy466-05, R&D Systems); IL-1β (88-7013, ThermoFisher Scientific); IL-12/IL-23p40 allele-specific (dy499-05, R&D systems), and TNF (BMS607, ThermoFisher Scientific). Immunoglobulin changes in murine serum (dilution 1:1000) were screened using the Ig Isotyping mouse uncoated ELISA kit (88-50630; ThermoFisher Scientific), as were intestinal tissue levels of IgA (dilution 1:10). All ELISAs were measured at optical density (OD) 450 nm using a Tecan Sunrise plate reader (Tecan Ltd; Reading, UK).

### 2.6. Immunoblot Analysis of A20 and IκBα Protein in Small Intestinal Tissue Lysates

RIPA-extracted intestinal tissue lysates (N = 3 mice per group) were loaded to nitrocellulose (100 ug protein) using a slot blot vacuum system for immunodetection of A20 (TNFAIP3) and IκBα protein. Membranes were first blocked with 5% *w*/*v* BSA for 2 h, followed by overnight incubation with primary antibodies in sterile PBS pH7.4 containing 0.5% BSA, 0.05% Tween 20. Primary antibodies used were anti-TNFAIP3 mouse monoclonal antibody, clone 59A426 at 1:200 dilution (#ab13597—Abcam; Cambridge, UK), and an anti-IκBα polyclonal antibody at 1:1000 dilution (#9242; Cell Signaling Technology). Secondary HRP-linked antibodies used were anti-mouse IgG1 (#7076) and anti-rabbit IgG (#7074), each at 1:2000 (Cell Signaling Technology). Chemiluminescence detection was performed using SuperSignal West Dura Extended Duration Substrate (ThermoFisher Scientific), with imaging and densitometry analysis on a ChemiDoc XRS+ system (BioRad; Hemel Hempstead, UK).

### 2.7. Data Analyses

Results are expressed as mean ± standard error of mean (SEM). Independent sample groups were first assessed for normality and equality of variances, and then as appropriate using either a one-way ANOVA or non-parametric Kruskal–Wallis test followed by pairwise comparisons of treatments. Statistical analyses of datasets utilized Prism GraphPad v9.1.0 (GraphPad Software, San Diego, CA, USA) and StatsDirect v3.0.171 (StatsDirect Ltd.; Birkenhead, UK). Differences were considered statistically significant when *p* < 0.05.

## 3. Results

### 3.1. TNF-Induced NFκB-Dependent Serum Cytokine/Chemokine Responses Were Attenuated in IL-10-Deficient Mice

**Figure 1:**
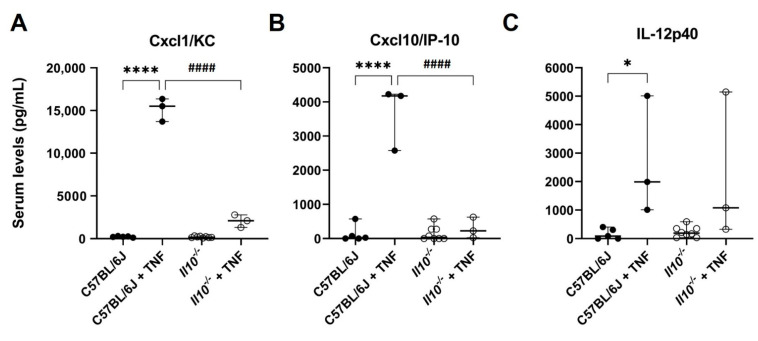
Attenuated serum levels of Cxcl1/KC and Cxcl10/IP-10 seen in IL-10-deficient mice following intra-peritoneal injection with TNF. Levels of (**A**) Cxcl1/KC, (**B**) Cxcl10/IP-10, and (**C**) IL-12p40 in serum of C57BL/6J (N = 5) and *Il10^−/−^* mice (N = 8) at resting levels, and those receiving i.p. injection of 0.33 mg TNF/kg body weight for 1.5 h (both genotypes N = 3). Serum was obtained from whole blood collected by cardiac puncture, with cytokines/chemokines (pg/mL) measured by ELISA. Significant differences compared to C57BL/6J control mice at rest, * *p* < 0.05, **** *p* < 0.0001; and significant differences from TNF-treated C57BL/6J mice, #### *p* < 0.0001 (ANOVA).

In this study, we used a well-established experimental model of systemic inflammation induced by TNF, which results in an increased intestinal damage. Intra-peritoneal injection of C57BL/6J mice (N = 3) with TNF (0.33 mg/kg body weight, for 1.5 h) resulted in markedly elevated serum levels of NFκB-dependent inflammatory mediators Cxcl1/KC (68.3-fold), Cxcl10/IP-10 (27.1-fold), and IL-12p40 (16.5-fold) above those resting levels detected in naïve C57BL/6J mice (N = 5); all *p* < 0.05 ANOVA (Figure 1). No significant differences in resting levels of all three cytokines were seen in naive *Il10^−/−^* mice (N = 8). However, a significantly attenuated TNF-induced response was observed for both Cxcl1/KC (9.3-fold change) and Cxcl10/IP-10 (2.2-fold) in serum of IL-10 deficient mice (N = 3), as compared to TNF-treated C57BL/6J mice (N = 3; both *p* < 0.0001 ANOVA). IL-12p40 levels in serum in response to TNF were not seen to be significantly attenuated in IL-10-deficient mice (Figure 1).

We also examined for any differences in serum levels of immunoglobulins, as well as for specific changes in κ and λ light chains. At rest, there was a significant impairment noted for IgA and the κ light chain in IL-10-deficient mice (both *p* < 0.01 ANOVA); Supplementary Information—Figure S1. Upon TNF treatment, no significant induction of serum immunoglobulins or light chains was observed in wild-type mice, but there was a noted absence of IgA (*p* < 0.01). Serum from TNF-induced IL-10-deficient mice was found to have significantly elevated levels of IgG1, and likewise, levels of both κ and λ light chains were both raised compared to untreated genotype controls (all *p* < 0.05); Supplementary information—Figure S1.

### 3.2. Dysregulated Expression of TNF in the Resting Small Intestine of IL-10-Deficient Mice

**Figure 2:**
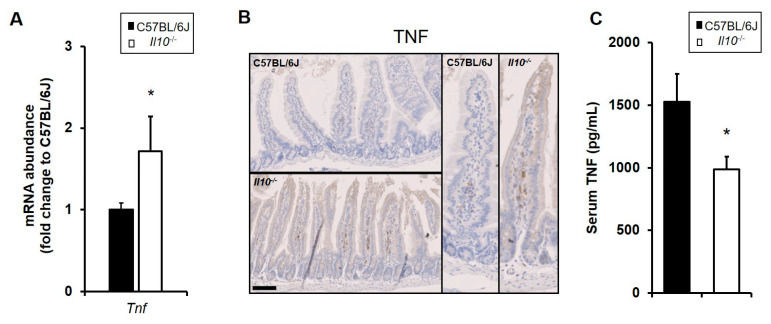
Dysregulated expression of TNF in resting small intestine of IL-10-deficient mice. C57BL/6J and *Il10^−/−^* mice under resting conditions were sacrificed, their sera collected, and tissue processed to assess for an abundance of mRNA and protein. (**A**) Enhanced *Tnf* mRNA levels were detected by qPCR in the small intestine of resting *Il10^−/−^* mice relative to C57BL/6J wild-type mice (both N = 3). (**B**) The C57BL/6J and *Il10^−/−^* small intestine was fixed in 4% *w*/*v* paraformaldehyde, with 4 μm sections processed and stained using antibodies against TNF. TNF staining appears only in lamina propria cells from *Il10*-deficient mice, but not in wild-type. Figures representative of N = 6 mice; three males and three females from each genotype; bar = 100 μm. (**C**) TNF levels in serum (pg/mL) were measured by ELISA, with lower levels being observed in IL-10-deficient mice under resting conditions (N = 8) compared to C57BL/6J (N = 5). Significant differences compared to C57BL/6J, * *p* < 0.05, Mann–Whitney U test.

Basal levels of *Tnf* mRNA transcript were measured in total RNA isolated from small intestinal tissue of C57BL/6J and *Il10*^−/−^ mice under resting conditions. *Tnf* gene expression was seen to be elevated by approximately two-fold in the small intestine of *Il10*^−/−^ mice, compared to wild-type controls (N = 3 mice; *p* < 0.05, Kruskal–Wallis test); see Figure 2A. To further investigate this observation, immunohistochemistry on tissue sections was performed (N = 6; three males and three females from each genotype). The intestinal epithelial cells in both strains did not show any staining, but TNF-positive cells were apparent in higher numbers within the lamina propria of *Il10*^−/−^ mice, likely lymphoid and myeloid cells (Figure 2B). TNF levels within serum were observed to be significantly lower in IL-10-deficient mice under resting conditions (*p* < 0.05, Kruskal–Wallis test; Figure 2C).

### 3.3. Impaired NFκB Activation and Inducible TNF Expression in the Small Intestine of IL-10-Deficient Mice

**Figure 3:**
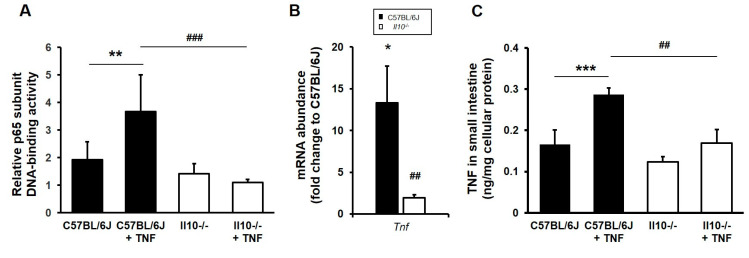
Aberrant NFκB activation and inducible TNF expression in the small intestine of *Il10*-deficient mice. C57BL/6J and *Il10^−/−^* mice were either kept at resting conditions or following intraperitoneal injection with recombinant murine TNF (0.33 mg/kg body weight). Mice were sacrificed after 1.5 h and tissue processed to assess for NFκB signal pathway activation and for abundance of *Tnf* mRNA and TNF protein. (**A**) NFκB ELISA of small intestine tissue lysates revealed TNF-induced p65[RelA] transcriptional activation in wild-type mice, which was impaired in the tissue of IL-10-deficient mice. (**B**) TNF-induced *Tnf* transcript levels were observed to be significantly diminished in *Il10*^−/−^ mice. (**C**) Similarly, tissue levels of TNF protein were not induced in intestinal mucosa of *Il10*^−/−^ mice 1.5 h post TNF injection. All data are presented as mean ± SEM, N = 3 mice. Significant differences for TNF-injected versus resting levels in C57BL/6J mice, * *p* < 0.05, ** *p* < 0.01, and *** *p* < 0.001; and TNF-injected *Il10^−/−^* versus TNF-injected C57BL/6J, ## *p* < 0.01 and ### *p* < 0.001 (Kruskal–Wallis test or ANOVA).

Following i.p. injection of mice with TNF, we examined the activation profile of the NFκB canonical pathway. Using small intestinal tissue lysates from naïve and TNF-injected mice, an ELISA was performed to assess p65[RelA] transcription factor activation. No significant difference in DNA-binding activity of p65 was observed under resting conditions in the *Il10*^−/−^ small intestine compared to wild-type tissue (Figure 3A). Upon i.p. injection of TNF, p65 activation was increased approximately two-fold in the wild-type small intestine (*p* < 0.01 Kruskal–Wallis test, N = 3 mice), but this increase in p65 activation was not observed in the TNF-treated, IL-10-deficient small intestine (*p* < 0.001, compared to TNF-injected, wild-type mice; N = 3); see Figure 3A. A significant upregulation of *Tnf* mRNA was observed in the C57BL/6J small intestine (13.3 ± 4.4-fold increase; Figure 3B) compared to non-injected controls (Figure 2A); *p* < 0.05 Kruskal–Wallis test; N = 3 mice). In contrast, there was an attenuated TNF-induced *Tnf* response observed in *Il10*^−/−^ mice (1.9 ± 0.36-fold increase compared to the TNF-injected C57BL/6J response; *p* < 0.01); see Figure 3B. The same defect was observed for TNF at the protein level, as measured by ELISA, in small intestine tissue lysates. Post i.p. injection of TNF, the level of TNF protein measured in the C57BL/6J wild-type small intestine was 0.29 ± 0.02 ng/mg total protein (compared to 0.17 ± 0.04 ng/mg total protein in untreated C57BL/6J controls; *p* < 0.001 ANOVA), with significantly attenuated levels seen in intestinal tissue lysates of TNF-induced, IL-10-deficient mice (0.17 ± 0.03 ng/mg total protein (N = 3, *p* < 0.01 ANOVA); Figure 3C.

**Figure 4:**
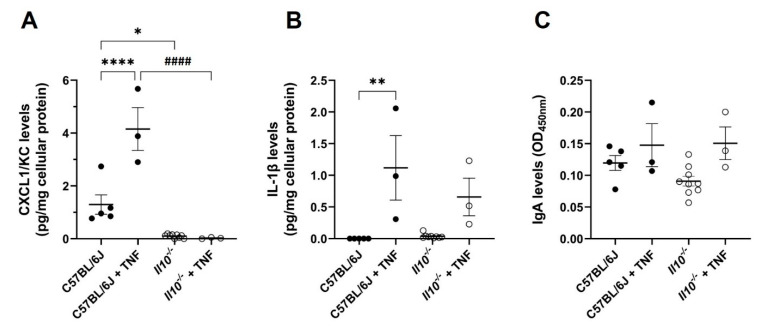
Attenuated intestinal tissue levels of NFκB-regulated pro-inflammatory cytokines, but not mucosal IgA, seen in IL-10-deficient mice following intra-peritoneal injection with TNF. Levels of (**A**) CXCL1/KC, (**B**) IL-1β, and (**C**) IgA in small intestinal tissue lysates of C57BL/6J (N = 5) and *Il10^−/−^* mice (N = 9) at resting levels, and those who received an i.p. injection of 0.33 mg TNF per kg body weight for 1.5 h (both genotypes N = 3). Cytokine/chemokine levels were measured by ELISA. Mucosal IgA was measured using the Ig Isotyping mouse uncoated ELISA. There were significant differences compared to C57BL/6J control mice at rest, * *p* < 0.05, ** *p* < 0.01, and **** *p* < 0.0001; and significant differences from TNF-treated C57BL/6J mice, ####* p* < 0.0001 (ANOVA).

We also observed that TNF induced significant proinflammatory IL-1β and Cxcl1/KC responses in wild-type C57BL6/J intestinal tissue (both *p* < 0.01 ANOVA, N = 3) when compared to non-injected controls (N = 5); see Figure 4. Cxcl1/KC levels in naïve *Il10^−/−^* mouse intestine (N = 9 mice) were significantly lower compared to those seen in wild-type intestine at rest (*p* < 0.05), and the response to TNF was markedly attenuated (*p* < 0.0001, N = 3); Figure 4A. TNF-induced IL-1β production was notably lower in IL-10-deficient intestine compared to TNF-injected wild-type controls but did not reach significance (Figure 4B).

Given observed changes in serum (monomeric) IgA in IL-10-deficient mice, and in response to i.p. injection of TNF, we measured mucosal (polymeric) IgA in intestinal tissue lysates but observed that levels were not significantly different in treatment groups (Figure 4C).

### 3.4. Altered A20 and IκBα Synthesis Observed in the Small Intestine of Il10^−/−^ Mice

**Figure 5:**
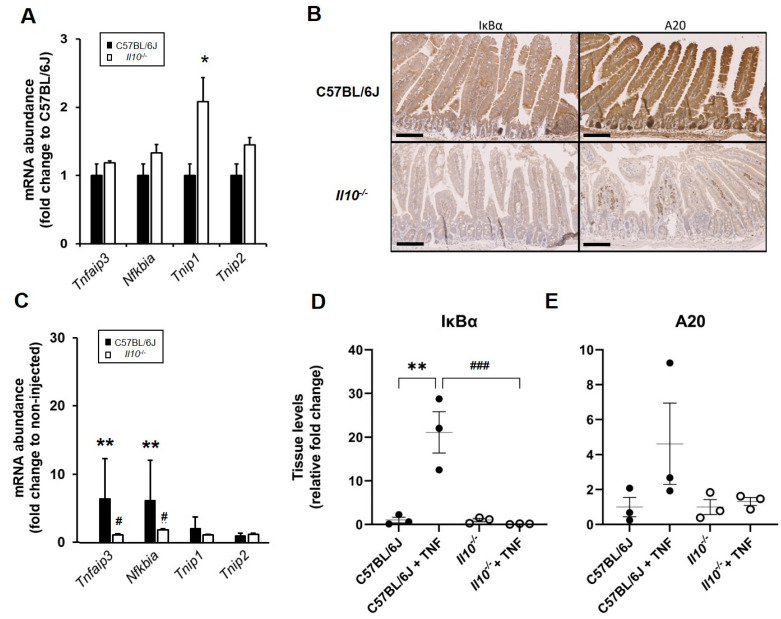
Dysregulated expression of key NFκB-targets in the small intestine of naïve and TNF-injected mice. C57BL/6J and *Il10^−/−^* mice were sacrificed at rest or following i.p. injection with recombinant murine TNF (0.33 mg/kg body weight). Tissue was dissected and processed for gene and protein expression analyses. (**A**) Expression of NFκB-target genes at rest in *Il10^−/−^* intestinal tissue (white bars) as assessed by qPCR. Data are presented as mean ± SEM, expressed as fold change to C57BL/6J (both N = 3 mice; significant difference compared to C57BL/6J, * *p* < 0.05 Kruskal–Wallis test). (**B**) Dysregulated A20 and IκBα protein expression in resting small intestine of *Il10*-deficient mice (as assessed by immunohistochemistry of 4% *w*/*v* paraformaldehyde-fixed, 4 μm microtomy tissue sections). Representative images from N = 6 mice; bar =100μm). (**C**) NFκB-target gene expression 1.5 h post-TNF injection of *Il10^−/−^* (white bars) compared to injected C57BL/6J mice (black bars); each N = 3 mice, with significant differences compared to non-injected controls ** *p* < 0.01, and to TNF-injected C57BL6/J # *p* < 0.01 (Kruskal–Wallis test). Intestinal tissue levels of (**D**) IκBα and (**E**) A20, expressed as relative fold change to respective genotype controls (N = 3 for all groups). There are significant differences compared to C57BL/6J * *p* < 0.05 and ** *p* < 0.01; and TNF-injected controls, ### *p* < 0.001 (ANOVA).

The defect we observed in RelA activation in response to TNF in IL-10-deficient mice prompted us to check further downstream targets of the NFκB pathway, including *Tnfaip3* and *Nfkbia*, encoding key inhibitory regulators of NFκB, i.e., A20 and IκBα, respectively. The basal transcription of *Tnfaip3* and *Nfkbia* genes from total RNA isolated from small intestinal mucosa was similar in both genotypes (Figure 5A). We also examined expression levels of *Tnip1* and *Tnip2* genes encoding the A20-binding inhibitor of NFκB proteins ABIN1 and ABIN2 known to suppress NFκB signaling [28]. At rest, only *Tnip1* showed higher levels of expression in the small intestinal mucosa of IL-10-deficient mice (2.1-fold increase) relative to wild-type tissue (*p* < 0.05; Kruskal–Wallis test, N = 3 mice); Figure 5A. This was followed by immunohistochemistry of 4% paraformaldehyde-fixed, paraffin-wax-embedded tissue sections from the small intestine of both strains using specific antibodies targeting A20 and IκBα (Figure 5B). Whilst A20 staining appeared to be unaffected in lamina propria cells of *IL10*-deficient intestinal mucosa compared to wild-type tissue, it was notable that staining was markedly reduced in *Il10*^−/−^ intestinal epithelial cells. Similarly, IκBα staining was almost undetectable in lamina propria of both strains, but it was severely reduced in *Il10*^−/−^ intestinal epithelial cells under resting conditions; see Figure 5B.

Post i.p. injection of TNF in wild-type C57BL/6J mice, we observed a significant upregulation in *Tnfaip3* and *Nfkbia* expression in the small intestine (6.5-and 6.2-fold increases in mRNA abundance, respectively; both *p* < 0.01 Kruskal–Wallis test). In contrast, TNF induction of *Tnfaip3* and *Nfkbia* in the intestinal tissue of *Il10*^−/−^ mice was defective (1.2- and 2.0-fold, respectively; both *p* < 0.05, compared to injected wild-type mice); Figure 5C. No such changes in *Tnip1* and *Tnip2* expression in the small intestine were observed in response to TNF injection of wild-type mice, with no differences observed in *Il10* knockout mice (Figure 5C). Immuno-detection of IκBα protein levels in intestinal tissue lysates revealed elevated endogenous levels present in response to the TNF treatment of C57BL6/J mice (21.1 ± 4.7-fold increase) relative to untreated C57BL/6J controls loaded and quantified to the same slot blot (1.0 ± 0.65; *p* < 0.01 ANOVA, N = 3); Figure 5D. A total absence of response to TNF was observed in the IL-10-deficient small intestine (*p* < 0.001 ANOVA, N = 3); see Figure 5D. A similar pattern was seen for A20 protein levels, although data did not achieve significance (all groups N = 3); Figure 5E.

Given that IL-10 receptor levels are important for IL-10 signaling, we also measured the expression of *Il10ra* in the small intestine in both genotypes under resting conditions and post i.p. injection of TNF (Supplementary information—Figure S2). Basal expression of *Il10ra* was 2.1-fold higher in the small intestine of *Il10*^−/−^ mice; *p* < 0.05 (N = 3, Kruskal–Wallis test). Post-TNF injection, both *Il10* and *Il10ra* were significantly upregulated in C57BL/6J mouse intestinal tissue, with significantly lower levels of TNF-induced *Il10ra* transcript detected in *Il10*^−/−^ mice; *p* < 0.05 (Kruskal–Wallis test).

## 4. Discussion

Interleukin-10 is considered a classical anti-inflammatory cytokine that downregulates pro-inflammatory pathways, including activation of NF*κ*B in immune cells such as macrophages and dendritic cells [29]. We recently showed that epithelium-derived IL-10 appears to be a positive regulator of NF*κ*B in vitro, supporting its role as a major regulator of intestinal homeostasis [24]. Here, in the current study, we utilized IL-10-deficient mice to evaluate further the role of IL-10 within the intestinal epithelium in a TNF-induced in vivo model of inflammation that impacts on intestinal homeostasis [30,31].

Naïve IL-10-deficient mice looked healthy and did not show any signs of inflammation, although they had lower TNF serum levels than the wild-type C57BL/6J controls. *Tnf* mRNA was higher in small intestine from resting *Il10^−/−^* mice, but immunohistochemistry on fixed tissue revealed that this was mainly due to a small increase in TNF-expressing cells localized within the lamina propria, possibly activated T cells or myeloid cells [32]. This is in agreement with previous reports that showed that *Il10*^–/–^ mice reared in germ-free conditions remained disease-free [33], and only inoculation with specific commensal bacterial strains induced colitis via an antigen-driven Th1 response [34,35]. Upon i.p. injection of TNF, wild-type mice responded with observed increases in serum levels of inflammatory chemokines, Cxcl1/KC and Cxcl10 and interleukin 12, the latter reflected by raised levels of the p40 subunit (IL-12p40). TNF has been reported to induce Cxcl10 production within epithelium via the canonical NF*κ*B pathway, as well as in the endothelium, again via activation of the same pathway [36]. The *Il10*-deficient mice showed a defective increase of serum Cxcl10 in response to TNF injection, implying that the source of the chemokine is probably from epithelial cells, and not from immune cells where IL-10 is observed to be a negative regulator of Cxcl10 [37]. Similarly, serum Cxcl1/KC levels were extremely low in samples obtained from TNF-treated, IL-10-deficient mice. In contrast, IL-12p40 serum levels were comparable between the two genotypes, ruling out IL-12p40 regulation by IL-10 that had previously been suggested in lipopolysaccharide-induced macrophages [38].

IL-10 is also known to regulate growth and differentiation of B lymphocytes [39,40] and induce class switch recombination (CSR) in both mice and humans [41,42,43,44]. We therefore examined the levels of immunoglobulins present within the serum, pre- and post-intraperitoneal injection of TNF. In naïve mice, amongst all the immunoglobulins examined, only serum IgA showed dramatic reduction in levels within IL-10-deficient mice, compared to wild-type C57BL6/J controls. We speculate that this is likely due to a loss of peritoneal B1-a subclass of B lymphocytes, a feature reported previously in *Il10*^−/−^ mice [45]. Normal IgA levels were observed in the intestinal mucosa and this is possibly due to the fact that within the intestine, TGF-β, and not IL-10, is the major critical switch factor that controls IgA class CSR [46,47].

TNF injection induced the transcription of *Tnf* in the intestinal epithelium of wild-type mice, a result similar to that which others, and our group, have reported previously [24,31,48]. There was clear impairment of *Tnf* mRNA induction in *Il10^−/−^* small intestinal tissue, supporting our earlier in vitro studies where we clearly showed defective TNF-induced transcriptional activation of the *Tnf* gene in IL-10-deficient intestinal epithelium [24]. Real-time qPCR results were further validated by the measurement of TNF protein levels, which showed a 50% reduction in intestinal tissue lysates taken from IL-10-deficient mice compared to the wild-type controls. These lysates were also utilized for measurement of RelA/NFκB activity, since the RelA/p50 complex is the most-studied heterodimer in the canonical pathway [49,50] and the RelA subunit contains a key transactivation domain responsible for its cellular function [51]. In small intestinal tissue from naïve C57BL/6J mice, RelA DNA-binding activity was detected at basal levels and induced by i.p. injection of TNF in vivo. Whilst RelA/NFκB activity did not appear to be significantly reduced in tissue from resting *Il10^−/−^* mice, its further activation in this transgenic model was completely abolished upon TNF injection. These results indicate that IL-10 deficiency has a clear in vivo effect on the canonical NFκB pathway activation in intestinal tissue, supporting earlier data obtained under in vitro culture conditions, as we reported previously [24]. We speculate that TNF induces intestinal epithelium IL-10 synthesis, which has an autocrine effect, and activates NFκB signaling via its own receptor. This prompted us to further examine the expression of known downstream targets and inhibitors of the NFκB pathway within intestinal tissue, and whether there is any clear IL-10 dependency. At rest, there was no significant difference seen in *Tnfaip3* and *Nfkbia* mRNA levels, but within the *Il10^−/−^* small intestinal epithelial cells at rest, A20 and IκBα protein levels, encoded by *Tnfaip3* and *Nfkbia,* respectively, were observed to be extremely low in this transgenic strain compared to C57BL/6J mice. When challenged via i.p. with TNF, lower levels of *Tnfaip3* and *Nfkbia* mRNA transcripts were observed within the small intestine of *Il10^−/−^* mice compared to intestinal tissue taken from TNF- injected C57BL/6J mice. This defective response was further confirmed by examining the protein levels of the related proteins, A20 and IκBα, respectively. These results agree with our previous observations in which both of these genes were similarly affected by endogenous IL-10 deficiency in resting and TNF-induced enteroid cultures [24]. A20 has been shown to be upregulated by TNF, acting in synergy with Abin1 to prevent intestinal inflammation by restricting intestinal epithelial death and preserving tissue integrity [52]. Therefore, its defective expression in gastrointestinal mucosa could contribute to the natural inclination of the *Il10^−/−^* mice towards intestinal inflammation. Expression of *Tnip1* and *Tnip2*, encoding for A20-binding-1 (Abin1) and A20-binding-2 regulators of NFκB (Abin2), were not substantially affected in this experimental model. TNF-induced upregulation of *Il10ra* was also impaired in the small intestine of *Il10^−/−^* mice and this is important since the IL-10Rα subunit is responsible for binding and assembly of the receptor, and therefore can affect downstream signal transduction [53].

## 5. Conclusions

In summary, our study has shown that RelA/NFκB activation within the intestinal mucosa is positively regulated by IL-10 in vivo, affecting the expression of downstream target genes and their encoded proteins important for tissue homeostasis. Spatial information on NFκB activation would be a useful future approach, i.e., to establish in which epithelial cell and/or immune cell types within intestinal tissue these events occur. Two of the downstream targets observed to be affected were known inhibitors of the classical NFκB signaling pathway, IκBα and A20 (TNFAIP3). This was a key finding we had previously demonstrated in vitro, using small intestine crypt stem-cell-derived enteroids deficient in IL-10 [24], highlighting clearly that endogenous IL-10 can act as a positive regulator of the canonical NFκB pathway. The data here, using a whole animal approach, further support the importance of the IL-10-canonical NFκB signaling pathway axis in regulating intestinal mucosa homeostasis and in response to inflammatory triggers. Importantly, defective classical NFκB activation in the absence of IL-10 could be responsible for the dysregulated cytokine/chemokine levels observed in both serum and intestine, at rest, and in response to inflammatory triggers, such as TNF. Further in vivo experiments utilizing recombinant stable IL-10 dimers [54] would prove useful to support future therapeutic intervention for IL-10-regulated inflammatory conditions.

## Data Availability

Data generated or analysed during this study are included in this published article and Supplementary information files.

## Supplementary Materials

The following supporting information can be downloaded at: www.mdpi.com/article/10.3390/biology11101377/s1. Figure S1: Serum levels of immunoglobulins seen in C57BL/6J and IL-10-deficient mice, at rest and following intraperitoneal injection of TNF. Figure S2: Aberrant IL-10 receptor alpha gene expression in the small intestine of mice following intraperitoneal injection of TNF.
