# Supplementary material for: Interleukin-10 Deficiency Impacts on TNF-Induced NFκB Regulated Responses In Vivo
Source: Biology (Basel). 2022 Sep 20;11(10):1377. doi: 10.3390/biology11101377 (PMC9598475; doi:10.3390/biology11101377)
Supplement: Supplementary file 1 [file biology-11-01377-s001.zip › Supplementary information - Figure S1 09-08-22 FINAL.pdf]

Interleukin-10 deficiency impacts on TNF-induced NFκB regulated responses *in vivo*

Stamatia Papoutsopoulou, Liam Pollock, Jonathan M. Williams, Maya M. L. F. Abdul-Mahdi, Reyhaneh Dobbash, Carrie A. Duckworth and Barry J. Campbell

Supplementary information – Figure S1

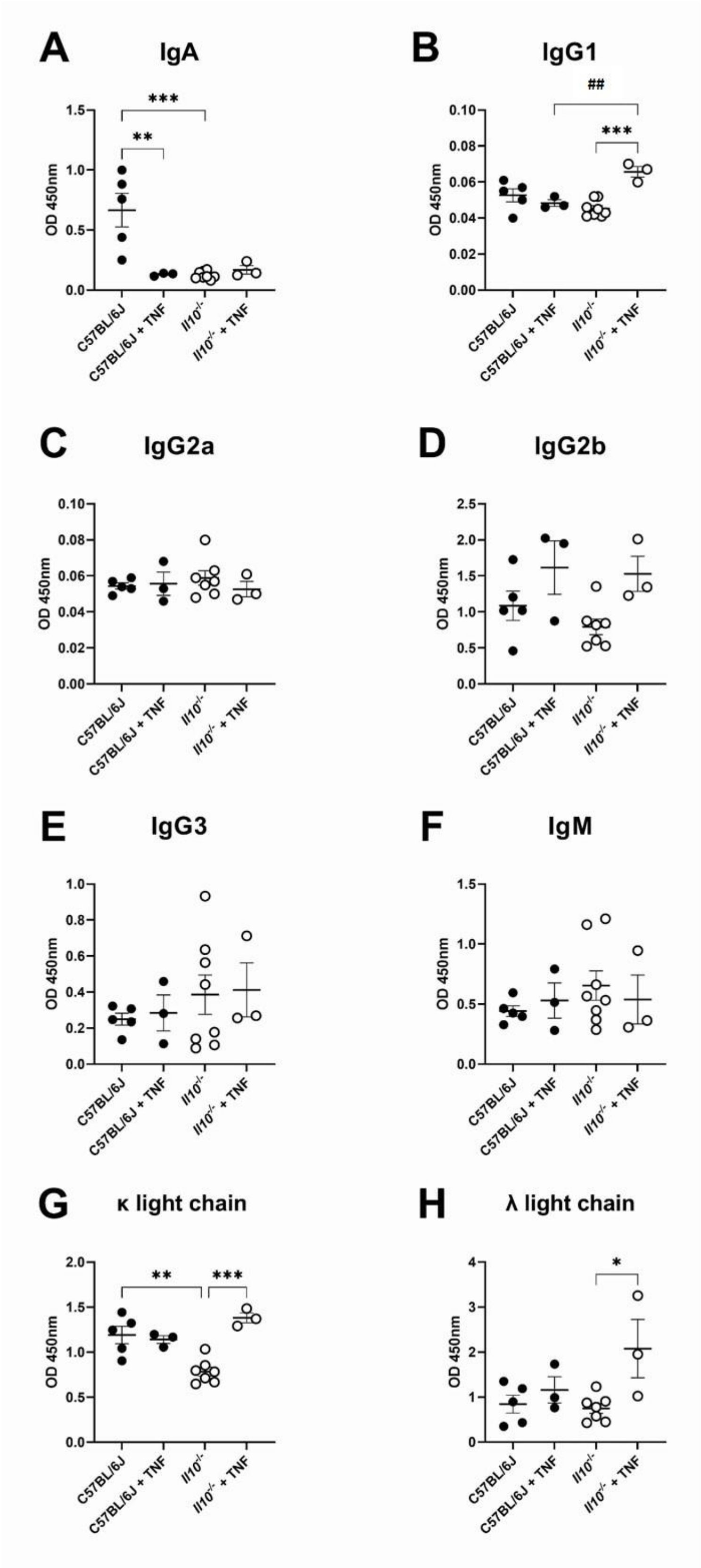

Figure S1: Serum levels of immunoglobulins seen in C57BL/6J and IL-10 deficient mice, at rest and following intraperitoneal injection of TNF. C57BL/6J (N=5) and *Il10*$^{-/-}$ mice (N=9) at rest, and those receiving an i.p. injection of recombinant murine TNF, 0.33 mg/kg body weight for 1.5h (both genotypes N=3), were sacrificed and blood collected by cardiac puncture. Levels of immunoglobulin (Ig) classes and two basic L (light) chains (κ and λ) in serum (1:1000 dilution) were screened by ELISA. Individual data presented as a scatter dot plot, including lines representing mean ± standard error of the mean (SEM). Significant differences compared to naïve C57BL/6J or *Il10*$^{-/-}$ mice at rest, **p*<0.05, ***p*<0.01 and ****p*<0.001; and significant differences from TNF treated C57BL/6J mice, ##*p*<0.01 (ANOVA, followed by Tukey's multiple comparisons test).
