# Supplementary material for: Interleukin-10 Deficiency Impacts on TNF-Induced NFκB Regulated Responses In Vivo
Source: Biology (Basel). 2022 Sep 20;11(10):1377. doi: 10.3390/biology11101377 (PMC9598475; doi:10.3390/biology11101377)
Supplement: Supplementary file 1 [file biology-11-01377-s001.zip › Supplementary information - Figure S2 09-08-22 FINAL.pdf]

Interleukin-10 deficiency impacts on TNF-induced NFκB regulated responses *in vivo*

Stamatia Papoutsopoulou, Liam Pollock, Jonathan M. Williams, Maya M. L. F. Abdul-Mahdi, Reyhaneh Dobbash, Carrie A. Duckworth and Barry J. Campbell

Supplementary information – Figure S2

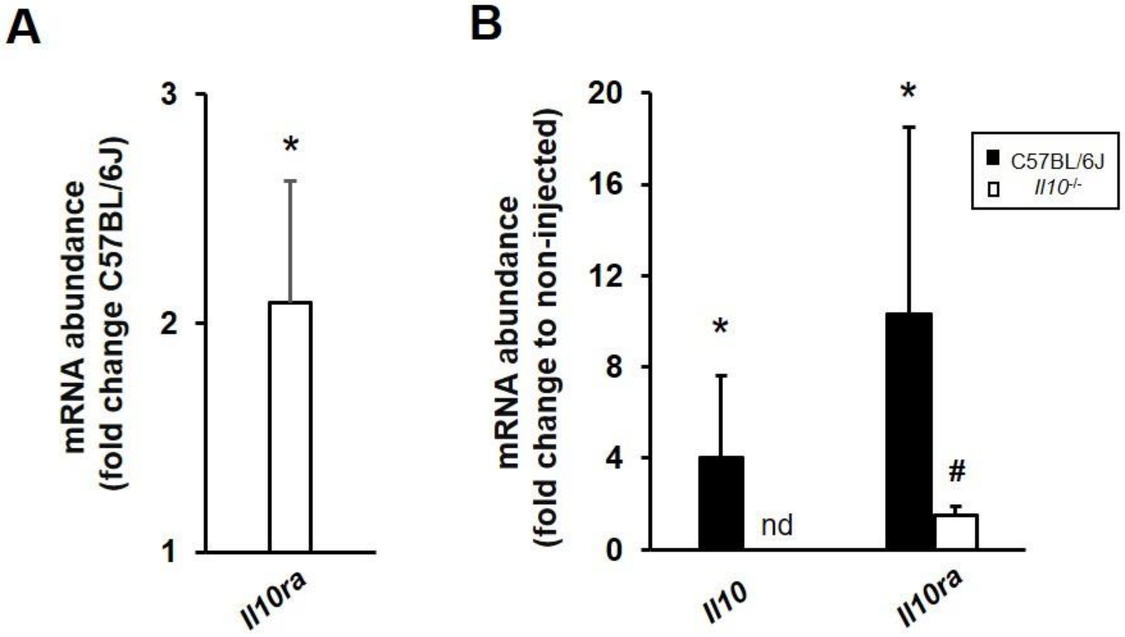

Figure S2. Aberrant IL-10 receptor alpha gene expression in the small intestine of mice following intraperitoneal injection of TNF. C57BL/6J and *Il10*$^{-/-}$ mice were either kept under resting conditions or injected i.p. with recombinant murine TNF (0.33 mg/kg body weight). Mice were sacrificed after 1.5h, tissue dissected and processed for qPCR analysis. Interleukin 10 (*Il10*) and IL-10 receptor alpha (*Il10ra*) mRNA levels observed in *Il10*$^{-/-}$ intestinal tissue (white bar); **(A)** at rest (expressed as relative fold change to naïve C57BL/6J mice), and **(B)** 1.5h post-TNF injection, *Il10*$^{-/-}$ *mice* (white bars) compared to C57BL/6J mice (black bars), expressed relative to non-injected mice. All data is presented as mean ± SEM, N=3 mice. Significant differences compared to naïve C57BL/6J mice, *p<0.05; and significant differences compared to TNF-treated C57BL/6J mice, #p<0.05 (Kruskal-Wallis test). nd = not detected.
